# Population expansion in the North African Late Pleistocene signalled by mitochondrial DNA haplogroup U6

**DOI:** 10.1186/1471-2148-10-390

**Published:** 2010-12-21

**Authors:** Luísa Pereira, Nuno M Silva, Ricardo Franco-Duarte, Verónica Fernandes, Joana B Pereira, Marta D Costa, Haidé Martins, Pedro Soares, Doron M Behar, Martin B Richards, Vincent Macaulay

**Affiliations:** 1 IPATIMUP (Instituto de Patologia e Imunologia Molecular da Universidade do Porto), Porto 4200-465, Portugal; 2 Faculdade de Medicina da Universidade do Porto, Porto 4200-319, Portugal; 3 Institute of Integrative and Comparative Biology, Faculty of Biological Sciences, University of Leeds, Leeds LS2 9JT, UK; 4 Department of Archaeology and Anthropology, University of Bristol, Bristol BS8 1UU, UK; 5 Estonian Biocentre and Department of Evolutionary Biology, University of Tartu, Tartu 51010, Estonia; 6 Department of Statistics, University of Glasgow, Glasgow G12 8QQ, UK

## Abstract

**Background:**

The archaeology of North Africa remains enigmatic, with questions of population continuity *versus *discontinuity taking centre-stage. Debates have focused on population transitions between the bearers of the Middle Palaeolithic Aterian industry and the later Upper Palaeolithic populations of the Maghreb, as well as between the late Pleistocene and Holocene.

**Results:**

Improved resolution of the mitochondrial DNA (mtDNA) haplogroup U6 phylogeny, by the screening of 39 new complete sequences, has enabled us to infer a signal of moderate population expansion using Bayesian coalescent methods. To ascertain the time for this expansion, we applied both a mutation rate accounting for purifying selection and one with an internal calibration based on four approximate archaeological dates: the settlement of the Canary Islands, the settlement of Sardinia and its internal population re-expansion, and the split between haplogroups U5 and U6 around the time of the first modern human settlement of the Near East.

**Conclusions:**

A Bayesian skyline plot placed the main expansion in the time frame of the Late Pleistocene, around 20 ka, and spatial smoothing techniques suggested that the most probable geographic region for this demographic event was to the west of North Africa. A comparison with U6's European sister clade, U5, revealed a stronger population expansion at around this time in Europe. Also in contrast with U5, a weak signal of a recent population expansion in the last 5,000 years was observed in North Africa, pointing to a moderate impact of the late Neolithic on the local population size of the southern Mediterranean coast.

## Background

Despite much recent research, the archaeology of North Africa remains enigmatic, with questions of population continuity *versus *discontinuity taking centre-stage. Key issues concern the identity of the bearers of the Middle Palaeolithic (or Middle Stone Age) Aterian industry (the age of which has recently risen dramatically from ~40-20,000 years ago (ka) to ≥115-40 ka [1,2]), and whether or not there was continuity between these and the later Upper Palaeolithic populations of the Maghreb. This question has become more urgent with the discovery that the Aterian is associated in Northwest Africa with a very early appearance of evidence for behavioural modernity, such as perforated *Nassarius *shell beads, use of ochre and bone tools, and long-distance exchange networks - preceding those of southern Africa and making it likely that the Aterian was carried by anatomically modern (rather than archaic) humans [3]. The fate of the populations using this industry, and their possible connection with others in Africa and with the group who dispersed out of Africa ~60 ka to populate the rest of the world, has naturally become a question of great interest.

Further debates have focused on the question of population replacement in the late Pleistocene and Holocene. The earliest Upper Palaeolithic industry in North Africa is the Dabban, limited to Cyrenaïca (a likely glacial refuge area [4]) and most likely dating from ~36-50 ka to ~20 ka [5]. Its similarities to Near Eastern Early Upper Palaeolithic industries have suggested an origin in the Levant [5], rather than locally in the Aterian, and similar blade industries have been found further south by 30 ka [1]. The Dabban was replaced by the Eastern Oranian (or Eastern Iberomaurusian) in Cyrenaïca possibly as early as ~18 ka [1], by which time the Upper Palaeolithic had extended eastwards for the first time along the coastal belt of the Maghreb, with the Iberomaurusian (or Oranian), the origins of which are similarly controversial, especially as it appears earliest in the Northwest. It began at the Last Glacial Maximum (LGM), ~22-20 ka, with a period of intensification in the Late Glacial ~15-13 ka, although recent work suggests a possible underlying industry as early as ~26 ka [1,6]. Similar debates concerning continuity *versus *replacement surround discussion of the widespread Epi-Palaeolithic Capsian industries, which saw expansion southwards from eastern Algeria into an increasingly humid Sahara in the early Holocene and sometimes associated with more gracile Mediterranean skeletal remains, and the subsequent emergence of the Neolithic [4,7-10].

The phylogeographic analysis of human mitochondrial DNA (mtDNA) has the potential to address questions such as these [11]. In particular, the mtDNA haplogroup U6 has a unique and highly distinctive distribution amongst human mitochondrial DNA lineages. It is found primarily in North Africa and the Canary Islands (albeit with secondary dispersals into Iberia and East Africa), with its highest frequency amongst Algerian Berbers (28%), and it has therefore been proposed to be linked to the ancestors of the indigenous Berber-speaking populations of North Africa [12-15]. Macaulay et al. [15] described U6 and its sister clade U5 as having evolved from a common ancestor in the Near East, approximately 50 ka; while U5 spread along the northern Mediterranean coast with the European Early Upper Palaeolithic, U6 dispersed along the southern coast, as far as Cyrenaïca, alongside the Dabban industry, ~40-50 ka, with a further expansion into Northwest Africa with the Iberomaurusian culture, ~22 ka. On this view, U6 evolved *en route *or within North Africa (as U5 evolved within Europe [11]), the presence of occasional derived U6 lineages in the Near East would signal more recent gene flow from North Africa [16].

These early studies were based only on information from hypervariable region I (HV-I) in the mtDNA control region and a small number of diagnostic RFLPs in the coding region, assayed in samples from several populations of North Africa and Iberia. In most of these regions, U6 frequencies are ~10% or less [17], and even appear absent from some Berber communities in Tunisia [18,19]. The major sub-haplogroup U6a (characterized by control-region transitions from the ancestor of haplogroup U at nucleotide positions 16172, 16219 and 16278) is highly dispersed, occurring throughout North Africa (and at low levels in the Near East and Iberia), and a further nested subclade, U6a1 (characterized by an additional transition at position 16189), follows a similar distribution.

By contrast, U6b (characterized by variants at 16172-16219-16311) has a more limited range to the northwest of North Africa, the north of the Iberian Peninsula and, as a nested derivative (U6b1, characterized by a transition at position 16163), in the Canary Islands [13]. In particular, the U6b1 lineage in the Canary Islands has been considered a founder lineage for the colonization of this archipelago by the Guanches (culturally very similar to Northwest Africans), ~2-3 ka [13], a conclusion supported by studies of ancient DNA [20]. Hence its arrival suggests itself as a potentially useful calibration point for the mtDNA molecular clock, although the archaeological evidence for the colonisation time is rather insubstantial [21]. In fact, the absence of U6b1 lineages anywhere outside of the Canary Islands (the few exceptions detected in Spain and in Americas being most readily explained as recent migrants from there), and the failure to detect immediate ancestors in North Africa, seem to point to the emergence of this clade within the Canary Islands - although probably soon after their colonization, as it is observed across several islands.

Maca-Meyer et al. [22] performed the first study of complete U6 mtDNA sequences (with 14 samples), defining a new U6 sub-haplogroup, U6c (characterized by the HV-I transition motif 16169-16172-16189), which was even more geographically restricted than U6b - limited to the west of North Africa and, as a derivative (U6c1, with an additional 16129 substitution), in the Canary archipelago. Using coding-region age estimates as maximum limits for radiation times, they proposed that the proto-U6 spread from the Near East to North Africa ~30 ka, alongside the Iberomaurusian industry, with U6a reflecting an African re-expansion from the Maghreb *eastwards *in Palaeolithic times, and U6a1 a further reverse movement from East Africa back to the Maghreb, possibly coinciding with the probable Afroasiatic linguistic expansion. The clades U6b and U6c, restricted to West Africa, had more localized expansions; they argued that U6b reached Iberia at the time of the diffusion of the Capsian culture in North Africa.

However, a larger study by Olivieri et al. [23] was closer to the earlier interpretation of Macaulay et al. [15]. They confirmed the origin of U6, or at least that of its immediate ancestor, in southwest Asia, with an ancient introduction (alongside haplogroup M1, and the Dabban industry) to North Africa *via *the Levant, possibly during the Greenland Interstadial 12, from ~44-48 ka. They reaffirmed that the various U6 sub-groups originated in the southern Mediterranean area, dispersing subsequently to East Africa.

Coalescence time estimates for U6 and it subclades have varied considerably amongst these studies. Yet these are critical for studies of prehistoric dispersals, since reliable estimates can bracket the timing of demographically significant events. For example, a regionally-specific clade may have arisen from a migration event on the edge in the tree leading to that clade, and if the diversification has then arisen *in situ *rather than prior to the presumed founder event, the estimate of the time to the most recent common ancestor (TMRCA) can provide a minimum bound on the age of the migration event (motivating the "founder analysis" [24]). However, success rests on a number of requirements, principally that the phylogeny can be well-estimated and the molecular clock that converts genetic differences into time depth is well-calibrated [25]. Considerable progress has recently been made on both of these fronts by more sophisticated analyses of the richer data source provided by mtDNA complete sequences.

With respect to the molecular clock, there are many factors leading to uncertainty. There is the wide variation in positional mutation rates and violations of the independence of mutations at different positions. Obvious regions are the paired stems of rRNAs and tRNAs [26], which some authors remove from the analysis [27], but there are other locations in the mtDNA molecule which can also present a secondary structure related with a functional role [28]. There is the problem of multiple hits and saturation, leading to the curious observation that the total proportion of control-region polymorphisms in the African branches of the tree is lower than in the non-African ones. Selection is also an important issue, with a higher frequency of replacement substitutions in the younger branches of the human mtDNA phylogeny compared to the more internal branches [29-31]. Kivisild et al. [30] advocated the use of only synonymous diversity for estimation of the TMRCA, which is problematic for age estimations in young lineages, while Soares et al. [31] implemented a correction for the purifying selection effect on the mutation rate estimated for the entire molecule.

The choice of calibration points is also an important issue. Traditionally, an outgroup is used, where the split time with the human lineage can be assigned in some way. For humans, the closest one is that corresponding to the human-chimpanzee split, for which the fossil evidence is controversial and which is in a time frame very distant from the TMRCA of the mtDNA of *Homo sapiens*, rendering the application of a strict clock problematic [32]. One recent analysis additionally used the chronometric ages of the available Neanderthal sequences as calibration points [33]. A strategy of multiple calibration points in conjunction with relaxed-clock methods, where the rate is allowed to vary among branches in the tree [34] is appealing, but this has been hard to implement in the human tree because of unavailability of secure multiple calibration points. Bandelt et al. [25] advocate that calibrated radiocarbon dates in favourable pioneer-settlement situations with a well-defined founder mtDNA scenario and a rich archaeological record could be used for calibration purposes, but consensus for both radiocarbon dates and founder mtDNA lineages are far from being achieved in most known settlement situations. Endicott and Ho [27] applied an internal calibration to the human mtDNA tree by specifying priors on the ages of three nodes in the tree associated with demographic signals: the entry into Australia and New Guinea by establishing a minimum of 40 ka for haplogroup P; and the post-Last Glacial Maximum expansion of haplogroups H1 and H3 (unfortunately suggested as 18 ka). This internal calibration was performed using a Bayesian approach with the software BEAST [35], and resulted in a substitution rate 1.4 times higher than that resulting from the human-chimpanzee calibration. BEAST also, however, allows a reconstruction of effective population size through time, by using the Bayesian skyline plot (BSP [36]), based on a coalescent model analysed by Markov Chain Monte Carlo sampling. BSPs do not require a pre-specified parametric growth model as do other methods and, although designed for use with population data, they have also been used to attempt parameter estimation from haplogroup data with some apparent success [37].

Here, we analyse an additional 39 complete U6 genomes, from the full range of the U6 geographic distribution, including the Near East, Iberia and the Canary Islands. This has enabled us to construct a phylogenetic tree including 89 U6 genomes in total, and to re-evaluate the demographic history of the haplogroup, and its role in North African prehistory, in the light of the recent developments in the calibration of mutation rates. A comparison tree was inferred for 141 U5 sequences from the literature, allowing us to test the use of four alternative internal calibration points (the settlement of the Canary Islands, the settlement of Sardinia and its internal population re-expansion, and the split between haplogroups U5 and U6 around the time of the first modern human settlement of the Near East) against the recently developed complete genome clock with a correction for purifying selection.

## Methods

### Samples

Based on information from HV-I that allowed the general classification into haplogroups, we selected 39 U6 individuals for complete sequencing: 22 from Portugal, most of them included in a published Portuguese database [38] and some new data; 5 from the Canary Islands; 1 from Morocco; 2 from a sample of 583 Ashkenazi Jews (of Polish and Russian ancestry [39]), 7 from 1143 non-Ashkenazi Jews (of Turkish, Bulgarian, Moroccan, Tunisian, and Ethiopian ancestry), and 2 from 253 Near Eastern Palestinians. Except in the case of the Canary Islands dataset, where we selected 5 U6b1 samples randomly, all the available U6 samples were sequenced from each of these datasets. The samples belonged to unrelated individuals, who gave informed consent for their biological samples to be used for mtDNA characterisation. The work complied with the Helsinki Declaration of Ethical Principles (59^th ^WMA General Assembly, Seoul, October 2008) and was approved by IPATIMUP Ethics Commission.

### Complete mtDNA sequencing and nomenclature

We amplified mtDNA using 32 overlapping fragments as described elsewhere [40]. After purification, we used the forward primers for the sequencing, and in some cases, also the reverse primers (in the presence of polycytosine stretches, when polymorphisms A574C and T16189C occur). We performed sequencing on a 3100 DNA Analyzer (AB Applied Biosystems, Foster City, CA), and the resulting sequences were read with SeqScape (AB Applied Biosystems, Foster City, CA) and BioEdit version 7.0.4.1 [41], by two independent investigators. In cases of ambiguous base calls, the PCR and sequencing reactions were repeated. Furthermore, the protocols for rechecking both haplogroup-defining polymorphisms, previously well-established in the literature, as well as private mutations were followed [42]. Mutations were scored relative to the revised reference sequence, rCRS [43], and numbers 1-16569 refer to the position of the mutation in that sequence. The 39 complete mtDNA sequences have been deposited in GenBank (Accession Numbers HQ651676-HQ651714).

### Statistical analyses

For the U6 phylogeny reconstruction, besides the new 39 sequences obtained here, we used 50 complete sequences previously published [22,23,40,44-47], and some unpublished sequences deposited in GenBank from the Family Tree DNA Company (accession numbers provided in Additional File 1). We also inferred a U5 tree, from 141 complete sequences available in GenBank and listed in Additional File 1. Preliminary network analyses [48] led to a suggested branching order for the trees, which we then constructed most parsimoniously by hand. The software mtDNA-GeneSyn [26] was used to convert files.

For estimation of the TMRCA for specific clades in the phylogeny, we used ρ statistic frequentist, maximum likelihood and Bayesian phylogenetic analyses. We used the ρ statistic (mean sequence divergence from the inferred ancestral haplotype of the clade in question) with a mutation rate estimate for the complete mtDNA sequence of one transition in every 3,624 years [31], correcting for purifying selection by using the calculator provided with that paper. We estimated standard errors as in Saillard et al. [49]. We also obtained maximum likelihood (ML) estimates of branch lengths using PAML 3.13 [50], assuming the HKY85 mutation model with gamma-distributed rates (approximated by a discrete distribution with 32 categories). We converted mutational distance in ML to time using the same clock. Bayesian phylogenetic analyses were performed using BEAST 1.4.6 [35] with a relaxed molecular clock (lognormal in distribution across branches and uncorrelated between them) and the HKY model of mutation with gamma-distributed rates. For this analysis, we performed an independent mutation rate calibration using calibration points internal to the phylogeny, which could be associated with particular demographic events, as suggested before [27,32]. The four chosen calibration points were selected to be within the time scale of interest to this study. The dates of three of the calibration points were assigned a shifted exponential distribution in which the most recent age (indeed, the mode) for the given clade as well as the 95^th ^percentile were assigned.

- For the first point, we assigned a mode age of 45 ka and a 95^th ^percentile of 60 ka for the split between the U5 and U6 lineages, based on the hypothesis that some of the first settlers of Europe [51] were carrying haplogroup U that would later evolve into U5 [11]. Considering this, the age of the U5-U6 split would be a minimum of 45 ka old. Reliable archaeological dates are minimum estimates of a first settlement, justifying the use of the exponential distribution.

- The second calibration point with mode 2.3 ka and 95^th ^percentile 5 ka was the age of the U6b1 branch based on the archaeological date for the colonization of the Canary Islands. The first settlement of the Canary Islands is extremely uncertain and the archaeology of the islands is not extensive [21]. Furthermore, the fact that U6b is so uncommon in North Africa could mean that the populations that carried U6b into the Canary Islands disappeared by drift and U6b1 already existed outside the Canaries. Again an exponential distribution was used to capture this uncertainty.

- The third point with mode 8 ka and 95^th ^percentile 14 ka was within U5b3a1, a subclade of which, U5b3a1a, is found only on Sardinia [52]. Sardinia was permanently colonized at least 8 ka [53]. The colonization time most probably took place between the radiation of the Sardinian-specific branch (U5b3a1a) and its split point with mainland Europe (U5b3a1), indicating that the latter is necessarily higher than 8 ka [52]. Again the age of the clade may be substantially higher than 8 ka and therefore an exponential distribution was used.

- The fourth calibration point we used was an age estimate for the Sardinian U5b3a1a branch, assigned a normal distribution (truncated at zero) centred on 5.8 ka, with a standard deviation of 1,000 years. After the settlement of Sardinia, the population size was probably low (considering the long branch between U5b3a1 and the ancestor of U5b3a1a). It is tempting to identify the subsequent rather star-like radiation of U5b3a1a with a population expansion. The late Neolithic in Sardinia around 5-6 ka was a likely time of internal re-expansion and population increase [53] and we hypothesized that the age of U5b3a1a corresponds to this internal expansion. This interpretation gains strength from a signal elsewhere in the phylogeny: haplogroup M1 in Sardinia also presents a rather star-like clade [23] dating to 5.2 ka, based on ρ and the time-dependent clock.

BEAST uses a Markov-chain Monte-Carlo (MCMC) approach to sample from the posterior distributions of model parameters (branching times in the tree and substitution rates). Specifically, we ran 150,000,000 iterations, with samples drawn every 1,000 MCMC steps, after a discarded burn-in of 15,000,000 steps. We checked for convergence to the stationary distribution and sufficient sampling by inspection of posterior samples. We also obtained Bayesian skyline plots from BEAST and visualised them with Tracer v1.3 from posterior distributions of parameters run for 50,000,000 iterations (with samples drawn every 1,000 MCMC steps, after a discarded burn-in of 5,000,000 steps). We used a generation time of 25 years to rescale the vertical axis of the BSP to years. In addition, we forced the larger sub-haplogroups (U6abd, U6a, U6bd, U6b, U6c and U6d) to be monophyletic in the analysis, as the presence of fast-evolving positions (such as 16189 and 16311) leads to the reconstruction of diverse phylogenies, which would not be comparable with the one inferred using network analysis; MCMC updates which violated this assumption were immediately rejected.

To determine and visualise the geographical distribution of U6, U6a and U6bd, we constructed interpolation maps using the "Spatial Analyst Extension" of ArcView version 3.2 http://www.esri.com/software/arcview/. We used the "Inverse Distance Weighted" (IDW) option with a power of two for the interpolation of the surface. IDW assumes that each input point has a local influence that decreases with distance. The geographic location used is the centre of the distribution area from which the individual samples of each population were collected. The data used are listed in Additional File 1.

## Results and Discussion

The enlarged U6 phylogeny (Additional File 2) confirmed the principal sub-groups already identified [23], and did not reveal any novel ones. It highlights, however, that variation in HV-I can be misleading in regard to the branching structure, particularly when major splits in the phylogeny are supported only by mutations at hotspot positions, justifying Olivieri et al.'s caution in the naming of such clusters (their Fig. three). For example, the additional data now allows transitions at position 16189 in U6 to now be resolved into several events [23], showing that the old classification of "U6a1" based only on HV-I diversity [20], and unifying U6a1 with U6a2 and U6a3 in Olivieri et al. [23] is not reliable. The case for the postulated "U6a1" movement from East Africa back to the Maghreb advanced by Maca-Meyer et al. [20] is not favoured by our inferred phylogeny, as the 16189 transition does not identify non-monophyletic groups. Indeed, the only sub-clade which seems to have a preferred distribution in East Africa (three individuals from Ethiopia) falls within U6a2, with only coding-region diagnostic mutations at positions 6359 and 11204 (dating to 13.4 ± 4.0 ka).

**Figure 1:**
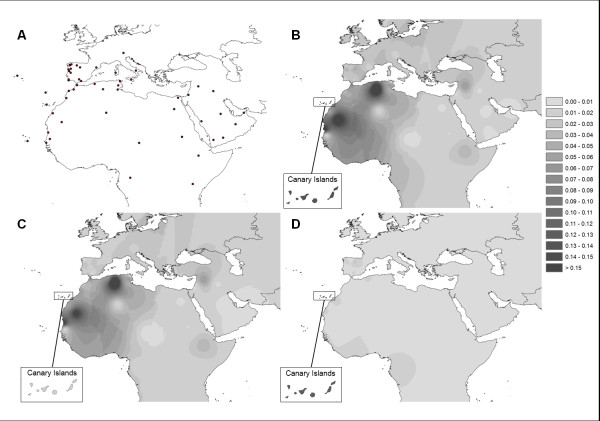
**Interpolation maps for U6 haplogroup**. (A) Map representing the centroids of sampling locations used for the spatial smoothing analyses of haplogroup frequencies (based on HV-I data sets). The resulting frequency maps are shown for U6 (B), U6a (C) and U6bd (D).

We display the geographic distribution of the frequencies of U6 and its major sub-clades across Europe, North Africa, the Arabian Peninsula and the Near East using HV-I data. Figure 1 confirms that U6a occurs most frequently in the west of North Africa, with two main peaks in Mauritania and Mozabites, most probably due to genetic drift, which is especially strong in the latter. U6bd (mostly likely U6b, since U6 d is very rare and cannot be distinguished from U6b on the basis of HV-I diversity) is restricted to the Canary Islands and to a few instances in North Iberia.

The estimated ages for the various U6 groups (Table 1) obtained using the internal calibration points have a mean ratio of 0.92 (for ρ) and 0.95 (for ML) relative to dates using the Soares et al. [31] mutation rate, corrected for purifying selection. Most of the major U6 subclades coalesce at times between the Last Glacial Maximum and the early Holocene, and the age of U6 as a whole is ~37 ka, but with a large 95% confidence range encompassing roughly 25-50 ka, due to the small number of early branching events.

**Table 1:** Age estimates for U6 and its major subgroups.

	A	B	C
U6	34,017 [23,533-44,908]	36,576 [24,876-48,764]	33,523 [24,543-46,863]

U6a	25,176 [19,696-30,784]	24,206 [19,969-28,521]	22,746 [16,873-29,418]

U6a1	17,903 [10,561-25,515]	19,551 [13,859-25,398]	19,839 [12,818-27,068]

U6a2	19,490 [12,420-26,800]	18,508 [13,477-23,664]	14,412 [10,460-18,752]

U6a3	20,115 [10,947-29,689]	16,767 [11,690-21,975]	14,767 [10,676-19,482]

U6a4	10,610 [3,182-18,361]	9,862 [3,860-16,077]	7,545 [3,134-13,768]

U6a5	13,370 [7,089-19,867]	13,467 [7,952-19,148]	12,263 [6,634-16,987]

U6a6	10,610 [3,182-18,361]	9,817 [3,816-16,030]	8,385 [3,297-14,515]

U6a7	26,541 [14,002-39,758]	22,323 [17,567-27,180]	22,154 [16,029-29,018]

U6b	12,304 [5,135-19,761]	11,816 [6,689-17,080]	13,915 [9,200-19,255]

U6b1	4,225 [1,029-7,488]	3,871 [1,246-6,540]	4,793 [2,877-6,950]

U6c	11,526 [4,263-19,090]	11,587 [4,406-19,063]	12,094 [5,317-17,785]

U6d	14,167 [6,473-22,181]	14,438 [8,190-20,894]	13,625 [9,181-18,466]

(A) Estimates based on ρ using the mutation rate of Soares et al. [31] for the complete sequence and a correction for purifying selection, with approximate 95% confidence intervals; (B) Maximum likelihood estimates using the mutation rate of Soares et al. [31] for the complete sequence and a correction for purifying selection, with approximate 95% confidence intervals; (C) Bayesian posterior medians of the ages based on the internal calibration points, with 95% highest posterior density intervals.

The posterior medians and 95% intervals for the ages of the points used for calibration were: for the U5-U6 split, 46,222 years [45,000-50,293]; U5b3a1, 9,825 years [8,000-12,714]; U5b3a1a, 5,850 years [4,953-6,743]; and U6b1, 4,835 years [2,958-7,064]. The posterior distributions for the oldest points agree better with the prior dates than the youngest point of U6b1, indicating that the Bayesian estimates will readapt the priors according to the existing information in the tree. This is most obvious in the case of the age of U6b1, suggesting that either the first settlement of the Canary Archipelago was earlier than the (admittedly weak) existing archaeological evidence indicates [21] or that U6b1 had already arisen prior to colonisation and has since drifted to extinction in the northwest African source, or at least has yet to be sampled. The estimates using the rate of Soares et al. [31] are both somewhat younger, suggesting that the Bayesian calibration (with only one calibration point in this range) may be failing to correct adequately for purifying selection at this time depth.

It is also worth pointing out that the age of haplogroup U reported here (the U5-U6 split at around 46 ka) is slightly lower than the age of U reported recently using ρ and ML and the complete sequence clock corrected for purifying selection [11,31] which tended to be > 50 ka. In fact, a ρ estimate of 44,145 years [32,460-56,254] is obtained using only the U5-U6 data with the Soares et al. rate; so the age of U in the Bayesian analysis would most probably have been higher if all the sub-branches of U had been included. The ages of U5b3a1 and U5b3a1a were 11,912 [3,456-20,777] years and 4,128 years [2,016-6,269] respectively. The comparison of the posterior mean for the ages in Bayesian with the ρ estimates for the four points indicate a mean ratio of 1.1 higher in the Bayesian analysis, in contrast to the trend obtained in the U6 ages described above. The Bayesian and ρ estimates overlap.

A further check of the clock is the age of U5a2a [54], since a single HV-I sequence belonging to this clade has been obtained from an ancient skeletal sample dated by radiocarbon to 7.8 ka [55]. The date obtained for U5a2a with the internal calibration was 8,045 years [4,391-13,184], concordant with the minimum possible age of the clade.

**Figure 2:**
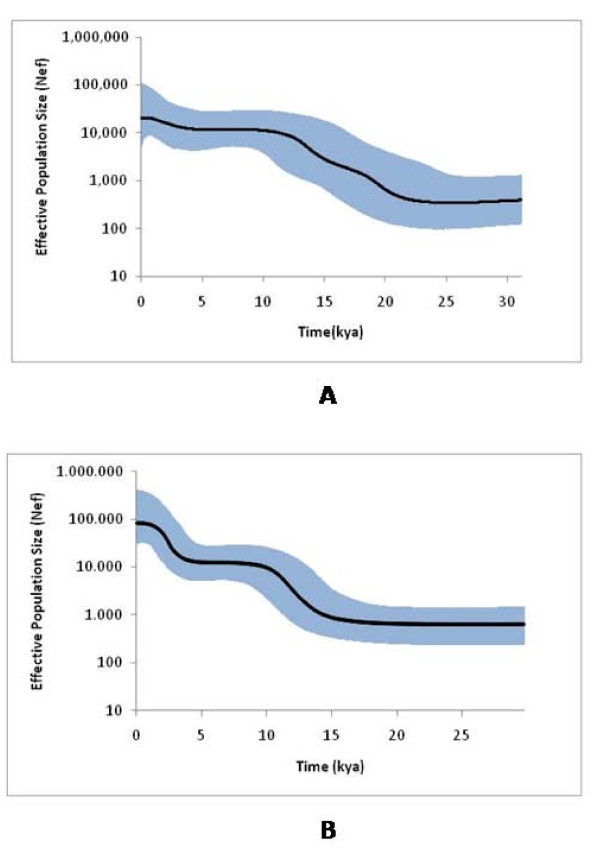
**BSPs of median effective population size through time for U6 (A) and U5 (B), using the internal calibration of mutation rate**. The black line represents the posterior effective population size through time. The blue region is the 95% highest posterior density interval for effective population size at any time point. Effective population size is plotted on a log scale. A generation time of 25 years was assumed.

The BSPs (Figure 2) computed for the U6 and U5 haplogroups (based on the mutation rate obtained with the internal calibration) display quite interesting patterns. One must keep in mind that BSPs were developed to estimate (effective) population size through time from a random sample of sequences [56], and there is little doubt that haplogroups do not equate to physically separated populations. Even so, the signs of expansions displayed in the U5 and U6 phylogenies may reflect expansions of populations bearing these haplogroups (as well as others), located in Europe and North Africa, respectively (compare the approach of Atkinson et al. [37] in sub-Saharan Africa). Another model assumption, panmixia, seems inappropriate for U5 and U6 together, since they have evolved on opposite sides of the Mediterranean Sea. Thus it seems appropriate to compute the BSPs separately. We performed this using the information gleaned from the initial internal calibration on the joint U5/U6 data set (in order to exploit calibration points in both haplogroups).

The BSP for U5 shows a small effective population size from the time of its origin until a strong expansion led to a ~11-fold increase within a period of ~6 ka in the late Pleistocene/early Holocene (14.3-8.3 ka). Following this, the effective size again remained more or less constant till a second strong expansion occurred, leading to an increase of ~5-fold after 4.3 ka (we took the year 1600AD as the most recent time-point [56]). For U6, the pattern is slightly more complex. The initial effective size was somewhat larger (~1.6 times) compared with U5, and the initial expansion begins earlier, ~22.2 ka, with a more gradual expansion until ~10.2 ka, leading to a 3-fold increase. As with U5, there is a further expansion in the Neolithic, after 4.8 ka with a shallower increase of ~1.5-fold.

## Conclusions

The Bayesian evolutionary analyses conducted in this work, as well as the comparison between recently updated mutation rates and the improvement of the phylogenetic resolution for the mitochondrial haplogroup U6, enable us to revise our interpretation of the evolutionary history of haplogroup U6 and sketch a demographic prehistory for North Africa, which we can then compare with that of Europe. In particular, they allow us to address many of the archaeological questions raised in the introduction.

The recently revised archaeological dates for the Aterian industry of North Africa emphasize that the makers of this industry do not appear to have left any imprint in the maternal lineages of present-day North Africans. The oldest arrivals amongst extant mtDNAs appear to be the U6 and M1 lineages, which date to 36.6 (24.9; 48.8) and 25.4 (17.9; 33.1) ka respectively [31]. As with U5 in Europe [11], the arrival time could be older in each case, since the haplogroups appear likely to have arisen within the southern Mediterranean region from haplogroup U and M ancestors, making dating the arrival time very imprecise. Nevertheless, the estimates seem to match best the appearance of the Upper Palaeolithic Dabban industry in Cyrenaïca, as suggested before [15,23].

There is an intriguing further signal in the U6 data, witnessed by the Bayesian skyline plot. For the European haplogroup U5, which is one of the most ancient in Europe [11], we identified a strong expansion (an ~11-fold increase in effective population size) occurring in the Lateglacial period between the LGM and the beginning of the Holocene, followed by another large population expansion (~5-fold) after 5 ka, evidently associated with late Neolithic/early Bronze Age (rather than, for example, the early Neolithic expansion in Europe, which began ~8.5 ka). For U6, by contrast, the corresponding increases in effective sizes were less marked (~3-fold and ~1.5-fold, respectively), and the signal indicates that the expansion began earlier, ~22 ka. This coincides closely with the beginning of the Iberomaurusian industry in the Maghreb. These results therefore suggest that the Iberomaurusian was initiated by an expansion of modern humans of ultimately Near Eastern, carrying mtDNA haplogroup U6, who had spread into Cyrenaïca ~35-45 ka and produced the Dabban industry. The link back to the Near East and the European Early Upper Palaeolithic (which likely has the same source) may explain the suggested skeletal similarities between the robust Iberomaurusian "Mechta-Afalou" burials and European Cro-Magnon remains, as well as the case for continuity of the bearers of the Iberomaurusian industry from Morocco with later northwest African populations suggested by the dental evidence [57].

We can compare the U5 and U6 BSPs with the ones for geographic regions published in Atkinson et al. [56], inferred from a more random sample of mtDNA sequences observed in those regions - for which the model behind the BSP is, on the surface, a better match. Similar to the situation reported for sub-Saharan Africa [37], the picture that emerges from U5 and U6 appears to represent well the general demographic patterns observed in, respectively, Europe and North Africa (although the latter was combined the Near East).

This observation suggests that by investigating in depth U6 and U5, the oldest lineages present in North Africa and Europe, respectively, we are indeed receiving signals from the demographic pre-history of modern humans in these regions.

Aside from U6, North Africa was also the recipient of European, Near Eastern and sub-Saharan African lineages most of which most likely arrived in the Holocene. Haplogroups H1, H3 and V expanded in Iberia in the Lateglacial/postglacial [11,58-61], and evidently spread into North Africa from Iberia across the Gibraltar Straits, most likely in the early Holocene [62-65]. Although the postglacial Capsian industry appears to have originated in eastern Algeria, it is tempting to hypothesize a connection with the arrival of these new populations from southwest Europe. Intriguingly, although U5b1, which also expanded from southwest Europe in the Lateglacial, has not been seen in Moroccan Berbers, it has been identified amongst Algerian Berbers and Fulbe from Senegal, as well as Iberia, Italy and northern Eurasian Saami and Yakut [44].

Most sub-Saharan lineages observed in North Africa are presently difficult to date and probably arrived at various times, but the age of the sub-Saharan subclade L3e5 indicates its arrival in North Africa from the south ~7 ka, following its expansion in the immediate postglacial humid phase ~11.5 ka [66]. Other L3 lineages seem to have been introduced even in more recent times, during the slave trade initiated by the Arab conquest of North Africa [67]. The Near Eastern haplogroups J and T (and probably K) appear to be concentrated more towards the east [68], mirroring the higher densities of U6, H and V in the west [64]. These may reflect the spread of the Neolithic into North Africa from the Levant, but their phylogeography awaits detailed analysis.

## Authors' contributions

LP conceived the study, coordinated its design and drafted the manuscript. RFD, VF, JBP and MDC carried out the molecular genetic studies, participated in the sequence alignment and in the phylogeny reconstruction. MBR and HM revised the manuscript, in particular focusing on the archaeology of North Africa and interpretation of the genetic results against the archaeological background. NMS and PS performed the statistical analyses and interpreted results with LP, DMB, MBR and VM. All authors read and approved the final manuscript.

## Supplementary Material

Additional file 1 **List of complete U6 and U5 samples and HV-I samples**. List of complete U6 and U5 samples used in this work, and of HV-I samples for the spatial smoothing analyses. Click here for file

Additional file 2 **Phylogeny of the complete U6 mtDNA sequences**. Phylogenetic tree reconstruction for 89 complete U6 sequences. Integers represent transition; only when a suffix ("A", "G", "C" or "T") is appended is a transversion indicated. Underlined nucleotide positions appear more than once in the tree. The rCRS is indicated. Click here for file
