# Supplementary material for: Population expansion in the North African Late Pleistocene signalled by mitochondrial DNA haplogroup U6
Source: BMC Evol Biol. 2010 Dec 21;10:390. doi: 10.1186/1471-2148-10-390 (PMC3016289; doi:10.1186/1471-2148-10-390)
Supplement: Additional file 1 — List of complete U6 and U5 samples and HV-I samples. List of complete U6 and U5 samples used in this work, and of HV-I samples for the spatial smoothing analyses. [file 1471-2148-10-390-S1.DOC]

Supplementary Material 1

Table 1 – List of U6 complete sequences

| Sample | Origin | Observations | Reference |
| --- | --- | --- | --- |
| AF382008 | Morocco |  | Maca-Meyer et al. (2001) |
| AY275527 | Morocco | Berber | Maca-Meyer et al. (2003) |
| AY275528 | Canary Islands |  | Maca-Meyer et al. (2003) |
| AY275529 | Senegal |  | Maca-Meyer et al. (2003) |
| AY275530 | Spain |  | Maca-Meyer et al. (2003) |
| AY275531 | Mauritania |  | Maca-Meyer et al. (2003) |
| AY275532 | Spain |  | Maca-Meyer et al. (2003) |
| AY275533 | Canary Island |  | Maca-Meyer et al. (2003) |
| AY275534 | Morocco |  | Maca-Meyer et al. (2003) |
| AY275535 | Mauritania |  | Maca-Meyer et al. (2003) |
| AY275536 | Morocco | Berber | Maca-Meyer et al. (2003) |
| AY275537 | Canary Islands |  | Maca-Meyer et al. (2003) |
| AY882416 | Ethiopia |  | Achilli et al. (2005) |
| AY882417 | Dominican Republic |  | Achilli et al. (2005) |
| DQ523663 | Sardinia |  | Fraumene et al. (2006) |
| DQ856317 | Italy |  | Unpublished - Family Tree DNA |
| EF064317 | Morocco | Berber | Olivieri et al. (2006) |
| EF064318 | Italy |  | Olivieri et al. (2006) |
| EF064319 | Italy |  | Olivieri et al. (2006) |
| EF064320 | Italy |  | Olivieri et al. (2006) |
| EF064321 | Algeria |  | Olivieri et al. (2006) |
| EF064322 | Italy |  | Olivieri et al. (2006) |
| EF064323 | Ethiopia |  | Olivieri et al. (2006) |
| EF064324 | Nigeria |  | Olivieri et al. (2006) |
| EF064325 | Morocco | Berber | Olivieri et al. (2006) |
| EF064326 | Morocco | Berber | Olivieri et al. (2006) |
| EF064327 | Iraq |  | Olivieri et al. (2006) |
| EF064328 | Italy |  | Olivieri et al. (2006) |
| EF064329 | Italy |  | Olivieri et al. (2006) |
| EF064330 | Nigeria |  | Olivieri et al. (2006) |
| EF064331 | Tunisia |  | Olivieri et al. (2006) |
| EF064332 | Italy |  | Olivieri et al. (2006) |
| EF064333 | Tunisia |  | Olivieri et al. (2006) |
| EF064334 | Morocco | Berber | Olivieri et al. (2006) |
| EF064335 | Italy |  | Olivieri et al. (2006) |
| EF064336 | Italy |  | Olivieri et al. (2006) |
| EF064337 | Italy |  | Olivieri et al. (2006) |
| EF064338 | Italy |  | Olivieri et al. (2006) |
| EF064339 | France |  | Olivieri et al. (2006) |
| EF064340 | Spain |  | Olivieri et al. (2006) |
| EF064341 | Italy |  | Olivieri et al. (2006) |
| EF064342 | Morocco | Berber | Olivieri et al. (2006) |
| EF064343 | Ethiopian | Jewish | Olivieri et al. (2006) |
| EF064344 | Italy |  | Olivieri et al. (2006) |
| EU597562 | Algeria |  | Hartmann et al. (2009) |
| FJ460538 | Tunisia |  | Costa et al. (2009) |
| FJ939330 | Unknown |  | Unpublished - Family Tree DNA |
| FJ979865 | France |  | Unpublished - Family Tree DNA |
| GU366066 | Unknown |  | Unpublished - Family Tree DNA |
| GU433197 | Canada |  | Unpublished - Family Tree DNA |
| I1 | Turkey | Jewish | This work |
| I2 | Morocco | Jewish | This work |
| I3 | Poland | Jewish | This work |
| I4 | Russia | Jewish | This work |
| I5 | Morocco | Jewish | This work |
| I6 | Tunisia | Jewish | This work |
| I7 | Ethiopian | Jewish | This work |
| I8 | Palestinian |  | This work |
| I9 | Palestinian |  | This work |
| I10 | Bulgaria | Jewish | This work |
| I11 | Bulgaria | Jewish | This work |
| M1 | Morocco |  | This work |
| P1 | Portugal |  | This work |
| P2 | Portugal |  | This work |
| P3 | Portugal |  | This work |
| P4 | Portugal |  | This work |
| P5 | Portugal |  | This work |
| P6 | Portugal |  | This work |
| P7 | Portugal |  | This work |
| P8 | Portugal |  | This work |
| P9 | Portugal |  | This work |
| P10 | Portugal |  | This work |
| P11 | Portugal |  | This work |
| P12 | Portugal |  | This work |
| P13 | Portugal |  | This work |
| P14 | Portugal |  | This work |
| P15 | Portugal |  | This work |
| P16 | Portugal |  | This work |
| P17 | Portugal |  | This work |
| P18 | Portugal |  | This work |
| P19 | Portugal |  | This work |
| P20 | Portugal |  | This work |
| P21 | Portugal |  | This work |
| P22 | Portugal |  | This work |
| SE30 | Canary Islands |  | This work |
| SE40 | Canary Islands |  | This work |
| SE81 | Canary Islands |  | This work |
| SE89 | Canary Islands |  | This work |
| SE161 | Canary Islands |  | This work |

Table 2 – List of U5 complete sequences

| Sample | Origin | Reference |
| --- | --- | --- |
| AF381989 | Morocco (Berber) | Maca-Meyer et al. (2001) |
| DQ156212 | Spain | Montiel-Sosa et al. (2006) |
| DQ282511 | USA ("Hispanic") | Just et al. (2008) |
| AY339544 | Finland | Finnila et al. (2001) |
| EF177408 | Portugal | Pereira et al. (2007) |
| EF363686 | Unknown | Family Tree DNA |
| EF397754 | Unknown | Family Tree DNA |
| EU049814 | Unknown | Family Tree DNA |
| EU124886 | Unknown | Family Tree DNA |
| EU151864 | Unknown | Family Tree DNA |
| EU215455 | Unknown | Family Tree DNA |
| FJ460552 | Tunisia | Costa et al. (2009) |
| FJ460558 | Tunisia | Costa et al. (2009) |
| GU012633 | France: Roussay (French) | Family Tree |
| GU371930 | Canada (Ukrainian) | Family Tree |
| GU391321 | Finland (Finnish) | Family Tree |
| AY339523 | Finland | Finnila et al. (2001) |
| AY339527 | Finland | Finnila et al. (2001) |
| AY339528 | Finland | Finnila et al. (2001) |
| AY339529 | Finland | Finnila et al. (2001) |
| AY714003 | North India: Uttar Pradesh (Brahmin) | Palanichamy et al. (2004) |
| AY882398 | Adygei | Achilli et al. (2005) |
| AY882399 | Italy | Achilli et al. (2005) |
| EU007851 | North Asia: Koryak | Ingman and Gyllensten (2007b) |
| EU140330 | Unknown | Family Tree DNA |
| EU140332 | Unknown | Family Tree DNA |
| EU140744 | Unknown | Family Tree DNA |
| EU523128 | Unknown | Family Tree DNA |
| EU597499 | French | Hartmann et al. (2008) |
| EU597527 | Israel (Palestinian) | Hartmann et al. (2008) |
| EU597544 | Italy: Bergamo | Hartmann et al. (2008) |
| EU698951 | Unknown | Family Tree DNA |
| GQ368895 | Poland: Lodz (Polish) | Family Tree |
| GU206811 | Finland (Finnish) | Family Tree |
| GQ160809 | Scotland: Western Isles (Scottish) | Family Tree |
| DQ785296 | Unknown | Family Tree DNA |
| GU459066 | USA (Irish ancestry) | Family Tree |
| AF346988 | Italy | Ingman et al. (2000) |
| AY339530 | Finland | Finnila et al. (2001) |
| AY339531 | Finland | Finnila et al. (2001) |
| AY339532 | Finland | Finnila et al. (2001) |
| AY339533 | Finland | Finnila et al. (2001) |
| AY339534 | Finland | Finnila et al. (2001) |
| AY339536 | Finland | Finnila et al. (2001) |
| AY339538 | Finland | Finnila et al. (2001) |
| AY339542 | Finland | Finnila et al. (2001) |
| AY339543 | Finland | Finnila et al. (2001) |
| DQ156208 | Spain | Montiel-Sosa et al. (2006) |
| DQ156210 | Spain | Montiel-Sosa et al. (2006) |
| DQ156214 | Spain | Montiel-Sosa et al. (2006) |
| DQ902697 | Sweden: Vasterbotten (Sami) | Ingman and Gyllensten (2007a) |
| DQ902705 | Sweden: Vasterbotten (Sami) | Ingman and Gyllensten (2007a) |
| EU597535 | Europe | Hartmann et al. (2008) |
| EU926618 | English | Family Tree DNA |
| DQ523645 | Sardinia | Fraumene et al. (2006) |
| EF420876 | Unknown | Family Tree DNA |
| EU367993 | Unknown | Family Tree DNA |
| GU295665 | USA | Family Tree |
| FJ499497 | Russia: Smolensk (Crimean Tatar/Ruthenian) | Family Tree |
| FJ194437 | unknown | Family Tree |
| AY882400 | Italy | Achilli et al. (2005) |
| AY882401 | Spain | Achilli et al. (2005) |
| AY882402 | Italy | Achilli et al. (2005) |
| AY882404 | Finland (Saami) | Achilli et al. (2005) |
| AY882405 | Yakut | Achilli et al. (2005) |
| AY882406 | Finland (Saami) | Achilli et al. (2005) |
| AY882408 | Algeria (Berber) | Achilli et al. (2005) |
| DQ781338 | Unknown | Family Tree DNA |
| EF420877 | Unknown | Family Tree DNA |
| AY882409 | Italy | Achilli et al. (2005) |
| AY882410 | Italy | Achilli et al. (2005) |
| DQ661681 | USA (Native American Cherokee) | Family Tree DNA |
| AY882411 | Italy | Achilli et al. (2005) |
| AY882412 | Algeria (Berber) | Achilli et al. (2005) |
| AY882413 | Italy | Achilli et al. (2005) |
| AY882414 | Spain | Achilli et al. (2005) |
| AY882415 | Italy | Achilli et al. (2005) |
| DQ523650 | Sardinia | Fraumene et al. (2006) |
| EF419891 | Unknown | Family Tree DNA |
| EF420249 | Unknown | Family Tree DNA |
| EF459670 | Unknown | Family Tree DNA |
| EU182656 | Unknown | Family Tree DNA |
| EU244000 | Ireland (Irish) | Family Tree DNA |
| EU490797 | United Kingdom: Northern Ireland | Family Tree DNA |
| EU594543 | Unknown | Family Tree DNA |
| EU682506 | USA | Family Tree DNA |
| EU694385 | Ireland | Family Tree DNA |
| EU784076 | Finland (Finnish) | Family Tree DNA |
| GQ853200 | Scotland: Aberdeenshire | Family Tree DNA |
| GQ132188 | unknown | Family Tree DNA |
| FJ916904 | Denmark: Viborg | Family Tree DNA |
| FJ887848 | USA; Irish/Scandinavian origin | Family Tree DNA |
| FJ664616 | USA | Family Tree DNA |
| DQ523624 | Italy: Sardinia | Fraumene et al. (2006) |
| DQ523628 | Sardinia | Fraumene et al. (2006) |
| DQ523658 | Sardinia | Fraumene et al. (2006) |
| DQ523669 | Sardinia | Fraumene et al. (2006) |
| GQ129143 | Italy: Southern Italy | Pala et al. (2009) |
| GQ129144 | France: Southern France | Pala et al. (2009) |
| GQ129145 | Bosnia and Herzegovina | Pala et al. (2009) |
| GQ129146 | Croatia | Pala et al. (2009) |
| GQ129147 | Spain | Pala et al. (2009) |
| GQ129148 | Italy: Northern Italy | Pala et al. (2009) |
| GQ129149 | Italy: Sardinia | Pala et al. (2009) |
| GQ129152 | Italy: Sardinia | Pala et al. (2009) |
| GQ129154 | Italy: Sardinia | Pala et al. (2009) |
| GQ129156 | Italy: Sardinia | Pala et al. (2009) |
| GQ129157 | France: Southern France | Pala et al. (2009) |
| GQ129158 | USA | Pala et al. (2009) |
| GQ129159 | Italy: Central Italy | Pala et al. (2009) |
| GQ129160 | Morocco | Pala et al. (2009) |
| GQ129161 | Italy: Central Italy | Pala et al. (2009) |
| GQ129162 | Italy: Central Italy | Pala et al. (2009) |
| GQ129163 | USA | Pala et al. (2009) |
| GQ129164 | Estonia | Pala et al. (2009) |
| GQ129165 | Greece | Pala et al. (2009) |
| GQ129166 | Spain | Pala et al. (2009) |
| GQ129167 | Czech Republic | Pala et al. (2009) |
| GQ129168 | Italy: Central Italy | Pala et al. (2009) |
| GQ129169 | Germany | Pala et al. (2009) |
| GQ129170 | Italy: Sardinia | Pala et al. (2009) |
| GQ129171 | Spain | Pala et al. (2009) |
| GQ129172 | Italy: Southern Italy | Pala et al. (2009) |
| GQ129173 | Spain: Southern Spain | Pala et al. (2009) |
| GQ129174 | Iraq | Pala et al. (2009) |
| GQ129175 | United Kingdom | Pala et al. (2009) |
| GQ129176 | Germany: Southern Germany | Pala et al. (2009) |
| GQ129177 | Poland | Pala et al. (2009) |
| GQ129178 | Bulgaria | Pala et al. (2009) |
| GQ129180 | Italy: Central Italy | Pala et al. (2009) |
| GQ129181 | Italy: Central Italy | Pala et al. (2009) |
| GQ129182 | Spain | Pala et al. (2009) |
| GQ129183 | Italy: Central Italy | Pala et al. (2009) |

Table 3 – List of population samples for HV-I diversity used in the interpolation analyses.

| **Number of individuals** | | | |  |  |
| --- | --- | --- | --- | --- | --- |
| ***N*** | ***U6*** | ***U6a*** | ***U6bd*** | **Region** | **Reference** |
| 84 | 3 | 3 | 0 | Portugal (North) | González et al. (2003) |
| 78 | 1 | 1 | 0 | Portugal (Central) | González et al. (2003) |
| 137 | 0 | 0 | 0 | Portugal (South) | González et al. (2003) |
| 187 | 10 | 6 | 4 | Portugal (North) | Pereira et al. (2004) |
| 239 | 6 | 6 | 0 | Portugal (Central) | Pereira et al. (2004) |
| 123 | 2 | 2 | 0 | Portugal (South) | Pereira et al. (2004) |
| 155 | 3 | 3 | 0 | Azores (Western/Central) | Brehm et al. (2003) |
| 179 | 6 | 5 | 1 | Madeira | Brehm et al. (2003) |
| 92 | 2 | 1 | 1 | Spain (Galicia) | Salas et al. (1998) |
| 43 | 1 | 0 | 1 | Spain (Galicia) | González et al. (2003) |
| 49 | 1 | 1 | 0 | Andalusia | Plaza et al. (2003) |
| 118 | 1 | 1 | 0 | Catalonia (Spain) | Crespillo et al. (2000) |
| 61 | 1 | 1 | 0 | Leon (Spain) | Larruga et al. (2001) |
| 38 | 2 | 1 | 1 | Castile (Spain) | Larruga et al. (2001) |
| 65 | 1 | 0 | 1 | Andalusia (Spain) | Larruga et al. (2001)+Côrte-Real et al. (1996) |
| 62 | 1 | 0 | 1 | Sardinia (Eastern) | Fraumene et al. (2006) |
| 300 | 42 | 3 | 39 | Canary Islands | Rando et al. (1999) |
| 75 | 1 | 1 | 0 | Italy (Sicily) | Ottoni et al. (2009) |
| 92 | 1 | 1 | 0 | Italy (Basilicata, South) | Ottoni et al. (2009) |
| 59 | 1 | 1 | 0 | Italy (Bolzano, North) | Thomas et al. (2008) |
| 60 | 5 | 5 | 0 | Morocco (Berber) | Rando et al. (1998) |
| 32 | 2 | 1 | 0 | Morocco (non-Berber) | Rando et al. (1998) |
| 50 | 3 | 3 | 0 | Morocco (Souss Valley) | Brakez et al. (2001) |
| 18 | 2 | 1 | 0 | Morocco (Arabs) | Plaza et al. (2003) |
| 81 | 2 | 2 | 0 | Morocco | Harich et al. (2010) |
| 56 | 3 | 3 | 0 | West Sahara | Plaza et al. (2003) |
| 25 | 2 | 2 | 0 | West Sahara | Rando et al. (1998) |
| 30 | 6 | 5 | 0 | Mauritania | Rando et al. (1998) |
| 64 | 11 | 11 | 0 | Mauritania | González et al. (2006) |
| 86 | 24 | 24 | 0 | Ghardaia, Northern Algeria | Corte-Real et al. (1996) |
| 47 | 2 | 2 | 0 | Tunisia | Plaza et al. (2003) |
| 190 | 13 | 13 | 0 | Tunisia (Arabs) | Lotfi et al. (2009) |
| 63 | 5 | 4 | 1 | Tunisia (Skira and Kesra, Berber) | Lotfi et al. (2009) |
| 51 | 0 | 0 | 0 | Tunisia (Tunis) | Lotfi et al. (2009) |
| 68 | 1 | 1 | 0 | Egypt | Krings et al. (1999) |
| 118 | 1 | 1 | 0 | Egypt | Rowold et al. (2007) |
| 102 | 1 | 1 | 0 | Sudan | This work |
| 77 | 3 | 3 | 0 | Ethiopia | This work |
| 100 | 1 | 1 | 0 | Kenya | Brandstätter et al. (2004) |
| 48 | 2 | 1 | 1 | Senegal (Wolof) | Rando et al. (1998) |
| 119 | 1 | 1 | 0 | Senegal (Mandenka) | Graven et al. (1995) |
| 60 | 2 | 1 | 1 | Africa (Fulbe) | Watson et al. (1997) |
| 24 | 2 | 2 | 0 | Africa (Kikuyu) | Watson et al. (1997) |
| 10 | 2 | 2 | 0 | Africa (Songhai) | Watson et al. (1997) |
| 26 | 2 | 2 | 0 | Africa (Tuareg) | Watson et al. (1997) |
| 292 | 9 | 9 | 0 | Cabo Verde | Brehm et al. (2002) |
| 448 | 2 | 1 | 1 | Chad | Cerny et al. (2007) |
| 109 | 2 | 1 | 1 | Saudi Arabia (Southern Region) | Abu-Amero et al. (2008) |
| 289 | 2 | 1 | 1 | Saudi Arabia (Central Region) | Abu-Amero et al. (2008) |
| 67 | 1 | 1 | 0 | Saudi Arabia (Western Region) | Abu-Amero et al. (2008) |
| 29 | 2 | 0 | 2 | Bedouin (Saudi Arabia) | Di Rienzo and Wilson (1991) |
| 249 | 1 | 1 | 0 | Dubai | Alshamali et al. (2008) |
| 90 | 1 | 1 | 0 | Qatar | Rowold et al. (2007) |
| 50 | 1 | 1 | 0 | Yemen | Rowold et al. (2007) |
| 185 | 0 | 0 | 0 | Yemen | Cerny et al. (2008) |
| 49 | 3 | 3 | 0 | Syria | Richards et al. (2000) |
| 117 | 1 | 1 | 0 | Iraq | Richards et al. (2000) |
| 117 | 1 | 1 | 0 | Israel (Palestinian) | Richards et al. (2000) |
